# Genome-Wide Assessment of Avocado Germplasm Determined from Specific Length Amplified Fragment Sequencing and Transcriptomes: Population Structure, Genetic Diversity, Identification, and Application of Race-Specific Markers

**DOI:** 10.3390/genes10030215

**Published:** 2019-03-13

**Authors:** Yu Ge, Teng Zhang, Bin Wu, Lin Tan, Funing Ma, Minghong Zou, Haihong Chen, Jinli Pei, Yuanzheng Liu, Zhihao Chen, Zining Xu, Tao Wang

**Affiliations:** 1 Haikou Experimental Station, Chinese Academy of Tropical Agricultural Sciences, Haikou 570102, China; wubin520327@163.com (B.W.); tanlin7402@126.com (L.T.); funingma@163.com (F.M.); 18754382266@126.com (Y.L.); zhihaocheng1@gmail.com (Z.C.); znlay1994@163.com (Z.X.); 2 Tianjin Derit Seed Industry Co. Ltd., Tianjing 300384, China; zhangteng@derit.cn; 3 South Subtropical Crops Research Institute, Chinese Academy of Tropical Agricultural Sciences, Zhanjiang 524091, China; zouminghong@163.com; 4 College of Agriculture, Guangxi Vocational and Technical College, Nanning 530226, China; haihongchen2019@163.com; 5 College of Horticulture and Landscape Architecture, Hainan University, Haikou 570228, China; zsk8920@gmail.com; 6 Institute of Vegetable, Liaoning Academy of Agricultural Sciences, Shenyang 110161, China

**Keywords:** *Persea americana*, ecological races, SLAF-seq, transcriptomics, phylogenetic relationships

## Abstract

Genomic data is a powerful tool. However, the phylogenetic relationships among different ecological races of avocado remain unclear. Here, we used the results from specific length amplified fragment sequencing (SLAF-seq) and transcriptome data to infer the population structure and genetic diversity of 21 avocado cultivars and reconstructed the phylogeny of three ecological races and two interracial hybrids. The results of the three analyses performed (unweighted pair-group methods with arithmetic means (UPGMA) cluster, Principal coordinate analysis (*PCoA*), and STRUCTURE) based on single nucleotide polymorphisms (SNPs) from SLAF-seq all indicated the existence of two populations based on botanical race: Mexican–Guatemalan and West Indian genotype populations. Our results based on SNPs from SLAF-seq indicated that the Mexican and Guatemalan races were more closely related to each other than either was to the West Indian race, which also was confirmed in the UPGMA cluster results based on SNPs from transcriptomic data. SNPs from SLAF-seq provided strong evidence that the Guatemalan, Mexican, and Guatemalan × Mexican hybrid accession possessed higher genetic diversity than the West Indian races and Guatemalan × West Indian hybrid accessions. Six race-specific Kompetitive allele specific PCR (KASP) markers based on SNPs from SLAF-seq were then developed and validated.

## 1. Introduction

Avocado (*Persea americana* Mill.) is among the most economically important subtropical/tropical fruit crops worldwide and is native to Central America and Mexico [1]. Avocado is a highly variable species, and there are local variations that have resulted in different ecological races [2]. Three of these ecological races of avocado are widely recognized by horticulturalists: Mexican (*P. americana* var. *drymifolia*), Guatemalan (*P. americana* var. *guatemalensis*), and West Indian (*P. americana* var. *americana*) [1]. These three ecological races are distinguishable based on morphological, horticultural, and physiological traits [3,4]. The Mexican race is widely considered to be native to Central Mexico and adapted to a relatively cold climate, whereas the Guatemalan race is thought to be mainly distributed in the mid and high altitudes of Guatemala’s mountains and is similarly somewhat cold tolerant [1]. The West Indian race is indigenous to central and northern South America and was only introduced into the West Indies in post-Columbian times; this race is characterized by adaptation to warm and humid tropical lowland conditions [5]. Avocado has a protogynous pattern of flowering, which promotes outcrossing, and sterility barriers do not exist between or among the three racial types [1]. Therefore, most commercial avocado cultivars are interracial hybrids [1].

Previous phylogenetic research on these three ecological races has typically resulted in diffuse racial boundaries or genetic relationships. Studies that used morphological characters [6] and different molecular markers [7,8] clustered the Guatemalan and West Indian races together. However, other research using isozymes [9] and molecular markers [10,11,12,13] could differentiate between the races. Ashworth and Clegg [11] suggested that the Guatemalan race is more closely related to the West Indian race than to the Mexican race, whereas Gross-German and Viruel [13] found that the Guatemalan and Mexican races were more closely related to each other than either were to the West Indian race.

A strongly supported phylogeny of these three ecological races is not available because of a lack of abundant, informative genome-scale datasets. Next-generation sequencing technologies have recently facilitated the generation of a large amount of DNA sequences and could serve as a powerful tool for analyzing population genetics and phylogenomics and identifying varieties [14]. Specific length amplified fragment sequencing (SLAF-seq) was developed based on high-throughput sequencing technology, and this technology has several obvious advantages, such as high-throughput, high accuracy, low cost, and short cycles, and the sequencing results can be directly used for molecular marker development [15]. This technology has been successfully used for genetic mapping [16,17], genetic diversity [18], gene identification [19], and phylogenomics analyses [20]. Analysis of genetic variability within transcriptomes using single nucleotide polymorphisms (SNPs) has the potential to resolve phylogenies and evolutionary history [21,22].

Race-specific markers could be used to identify the races of unknown avocado accessions and manage avocado genetic resources for breeding programs and germplasm conservation; however, the utility of few race-specific markers has been validated [12,13]. Ashworth and Clegg [11] suggested that interracial hybridization events occurred more recently than previously thought, and there were fewer differences among the botanical races than were expected. In addition, the limited number of molecular markers identified in previous studies mostly failed to sufficiently distinguish differences among the different avocado genomes [12]. Consequently, few useful race-specific markers have been found.

Here, we used SLAF-seq and transcriptome approaches to gain insight into the population structure and genetic diversity of 21 avocado cultivars and to clarify the phylogenetic relationships of the three ecological races. Our other aim was to identify and apply race-specific markers, which will help to validate the racial origin of accessions of unknown race.

## 2. Materials and Methods

### 2.1. Sample Collection, DNA Extraction, and RNA Extraction

For SLAF-seq, three ecological races and two interracial hybrids were collected from the South Subtropical Crops Research Institute, Chinese Academy of Tropical Agricultural Sciences (CATAS-SSCRI; Zhanjiang City, Guangdong Province, China: latitude 21°16′ N, longitude 110°22′ E, and altitude 30 m above sea level) and Guangxi Vocational and Technical College (GVTC; Nanning City, Guangxi Province, China: latitude 22°29′ N, longitude 108°11 E′, and altitude 79 m above sea level). A total of 21 individual accessions (two Mexican, two Guatemalan, three West Indian, seven Guatemalan × Mexican hybrid, and seven Guatemalan × West Indian hybrid accessions) were collected for analysis (Table 1). In addition, the out-group consisted of one *Cinnamomum micranthum* accession collected from CATAS-SSCRI. Fresh leaves were dried with silica gel prior to DNA extraction. Genomic DNA was extracted using the protocol described by Ge et al. [23] with minor modifications.

For transcriptome research, fresh leaves, apical buds, stems, flowers, and roots were collected from mature, healthy-appearing individuals of the Mexican (Duke 7), Guatemalan (Reed), and West Indian (Simmonds) races, and Guatemalan × Mexican (Fuerte) and Guatemalan × West Indian (Beta and Tonnage) hybrids, and immediately frozen in liquid nitrogen prior to storage at −80 °C. The root samples of these accessions were derived from in vitro propagated seedlings [24]. Total RNA was extracted using a Plant RNA Kit (OMEGA Bio-Tek, Norcross, GA, USA).

To use race-specific markers to identify the race structure of unknown race avocado accessions, eight unknown race avocado accessions were collected for analysis (Table 1). These eight avocado accessions with superior quality horticultural characteristics were all selectively bred from chance seedlings of unknown parentage in the garden that contains avocado germplasm resources at GVTC. Fresh leaves were dried with silica gel prior to DNA extraction. Genomic DNA was extracted using the protocol as described by Ge et al. [23] with minor modifications.

### 2.2. SLAF-seq and SNP Analysis

SLAF-seq was carried out as described by Sun et al. [15], with a few modifications. Briefly, genomic DNA derived from six accessions was digested by HaeIII and RsaI restriction enzymes. Then 264–314 bp DNA sequences were selected as SLAFs and used for paired-end sequencing on the Illumina Highseq 2500 (Illumina, Inc., San Diego, CA, USA). Based on the individual barcode sequences, low-quality reads (quality scores <30) were eliminated. After individual barcode sequences were removed from each high-quality read, clean reads from the same accessions were mapped onto the *C. micranthum* genome sequence (https://www.ncbi.nlm.nih.gov/genome/?term=Cinnamomum) by using the Burrows–Wheeler Aligner [27]. Samtools v0.1.18 [28] and Genome Analysis Toolkit v3 (GATK3) [29] were used for SNP calling. A SNP data packet that was generated by integrating GATK and Samtools SNP calling with default parameters was deemed as considered high quality. Consequently, 701,352 SNPs remained for further analysis.

### 2.3. Transcriptome Sequencing andSNP Analysis

A total of 1 μg RNA per accession was used for RNA sequencing. RNA sequencing libraries were produced with an NEBNext^®^Ultra™ RNA Library Prep Kit for Illumina^®^ (New England Biolabs, Ipswich, MA, USA) according to manufacturer recommendations and index codes were added to the attribute sequences of each sample. PCR products were purified using the AMPure XP system, and library quality was evaluated on the Agilent Bioanalyzer 2100 system. The library preparations were sequenced on an Illumina Hiseq 2000 platform and paired-end reads were produced. Clean data were obtained by removing reads including adapter sequences, reads including poly-N, and low-quality reads derived from raw data. Moreover, Q20, Q30, GC-content, and sequence duplication values of the clean data were calculated. The two read files were independently established from all of the libraries/samples. Transcriptome assembly was achieved based on these two files in Trinity v2.5.1 [30] with min_kmer_cov set to 2 and all other parameters set to default values. Clean data were mapped back onto the assembled transcriptome. Samtools v0.1.18 [28] was used to classify reads, eliminate duplicated reads and combine the false alignment results of each sample. GATK3 [29] was applied to carry out SNP calling. Raw data were eliminated using the GATK method, with MQ <40.0 and QD <2.0. Consequently, 65,535 SNPs remained for further analysis.

### 2.4. Identification and Application of Race-Specific Markers

Race-specific SNP loci were rigorously filtered by three steps in this study. First, the SNP loci were retained when the frequency of their missing alleles was 0.00 among the 21 avocado accessions. Second, the remaining SNP loci were selected when the alleles were present at a frequency of 1.00 for a pure avocado race and missing from the other two pure races. The third step was broken into three components: (i) the remaining Mexican-specific SNP loci were selected when the alleles of the Mexican accessions were present in all Guatemalan × Mexican hybrid accessions (*n* = 7); (ii) the remaining West Indian-specific SNP loci were selected when the alleles of the West Indian accessions were present in all Guatemalan × West Indian hybrid accessions (*n* = 7); and (iii) the remaining Guatemalan-specific SNP loci were selected when the alleles of the Guatemalan accessions were present in most of the Guatemalan × Mexican and Guatemalan × West Indian hybrid accessions (*n* ≥ 11).

To validate and apply race-specific SNP loci, primers for the race-specific SNP loci were developed to amplify DNA fragments for KASParassays [31] and named PaWIsSNP, PaGsSNP, and PaMsSNP to indicate *Persea americana* and race-specific SNPs (West Indian, Guatemalan, and Mexican, respectively). KASPar oligos were purchased from Sangon Biotech (Shanghai, China), with primers that had normal FAM- or VIC-compatible tails (FAM tail: 5′ GAAGGTGACCAAGTTCATGCT 3′; VIC tail: 5′GAAGGTCGGAGTCAACGGATT 3′) and the target SNP in the 3′ end. The primer mix was used as described by KBioscience (http://www.kbioscience.co.uk) (46 μL dH_2_O, 30 μL common primer (100μM), and 12 μL of each tailed primer (100 μM)).SNP amplification was carried out in a thermal cycler in a 5 μL volume, which included 1× KASP Master mix, 10 ng of genomic DNA, and the SNP-specific KASP assay mix. The amplification conditions were the same for each SNP assay: hot start at 94 °C for 15 min, followed by 10 touchdown cycles at 94 °C for 20 s and 58–61 °C for 60 s (dropping 0.8 °C per cycle), and then 35 amplification cycles (94 °C for 20 s; 57 °C for 60 s). Data analysis was manually carried out on a Roche Lightcycler 480 v1.50.39.

### 2.5. Data Analysis

Phylogenetic analyses were carried out based on 701,352 SNPs from SLAF-seq and 65,535 SNPs from transcriptome sequencing. After SNP detection, each SNPs was used to infer genetic distance in TASSEL v5.0 (http://www.maizegenetics.net/tassel).

Linkage disequilibrium-based pruning was carried out to filter out SNPs that were in linkage disequilibrium with one another through PLINK’s indep-pairwise command (SNP window size: 50, SNPs shifted per step: 5, *r* *2* threshold: 0.2), subsequently 34,704 SNPs were retained for population structure analysis, which was performed in STRUCTURE v2.3.4 [32]. To obtain the optimal *K* value, *K* was set between1 and10, and three independent runs were performed for every *K* with a burn-in of 100,000 iterations, followed by 100,000 Markov Chain Monte Carlo iterations for every *K* value. Then, the delta *K* method described by Evanno et al. [33] was used to detect the optimal *K* value by structure Harvester v0.6.94 (http://taylor0.biology.ucla.edu/structureHarvester).The Principal component analysis (*PCoA*) of the 21 samples was carried out based on the 701,352 obtained SNPs in EIGENSOFT v6.0.1 [34].

Assessments of genetic diversity, including minor allele frequency (MAF), observed heterozygosity (*Ho*), expected heterozygosity (*He*), Nei diversity index (*Nei*), Shannon’s information index (*I*), and polymorphic information content (PIC) were calculated based on the 701,352 SNP dataset through Perl programming. An analysis of molecular variance (AMOVA) based on 701,352 SNPs and 10,000 permutations was carried out using Arlequin v3.11 [35].

## 3. Results

### 3.1. Sequence Assembly and SNP Detection

After SLAF library construction and Illumina sequencing, 5.39 Gb of clean reads that contained 49.69 Mb of reads were obtained. The average guanine–cytosine (GC) content was 40.80%, and the average Q30 ratio (bases with a quality score of 30, which indicated a 1% chance of an error and thus 99% confidence) was 96.27% (Table S1). A total of 252,898 polymorphic SLAFs were generated from 22 samples, with an average sequencing depth of 78.59× (Table S1). In total, 2,643,532 raw SNPs were identified from 252,898 polymorphic SLAFs, and 701,352 SNPs remained for further analysis after filtering the 2,643,532 raw SNPs. Descriptions of the 701,352 SNPs from SLAF-seqs of the 21 avocado accessions and one *C. micranthum* accession are provided in Table S2.

Transcriptome sequencing generated 32.44 Gb of clean reads. The GC percentage ranged from 46.22–46.57% across all six accessions and mean GC percentage was 46.42% (Table S3). The mean Q30 percentage ranged from 94.46–95.51% and overall mean Q30 percentage was 94.67% (Table S3), and 65,535 SNPs were left for further analysis after filtering. Descriptions of the 65,535 SNPs analyzed from the transcriptomes of the six avocado accessions are provided in Table S4.

### 3.2. Phylogenomic and Population Analysis by SLAF-seq Based on SNP Data

**Figure 1:**
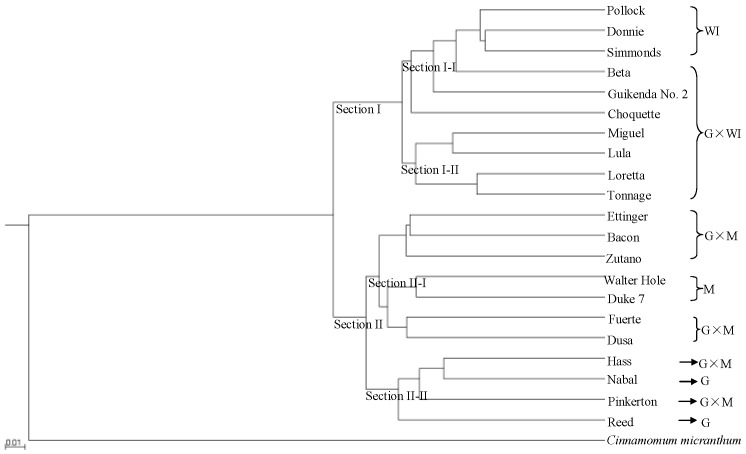
Rooted unweighted pair-group method with arithmetic means (UPGMA) phylogenetic tree of 21 avocado accessions based on 701,352 single nucleotide polymorphisms (SNPs) from specific length amplified fragment sequencing (SLAF-seq). M: Mexican, G: Guatemalan, WI: West Indian (interracial hybrids are indicated by ×).

UPGMA cluster analysis clearly resolved the two avocado sections (Figure 1), with 10 accessions classified as section I and 11 accessions classified as section II. Ecological race and interracial hybrids were clearly separated into sub-sections. Section I was composed of two sub-sections. Sub-section I-I included six accessions, three of which (Pollock, Donnie and Simmonds) were classified as West Indian, and the remaining three accessions (Beta, Guikenda and Choquette) were classified as Guatemalan × West Indian hybrids. In sub-section I-II, four Guatemalan × West Indian hybrids (Miguel, Lula, Loretta and Tonnage) clustered together. Section II was also composed of two sub-sections. Sub-section II-I included the two Mexican accessions (Walter Hole and Duke7) and five Guatemalan × Mexican hybrid accessions (Ettinger, Bacon, Zutano, Fuerte and Dusa). The two Guatemalan accessions (Nabal and Reed) and two Guatemalan × Mexican hybrid accessions (Hass and Pinkerton) clustered together as sub-section II-II. Table S5 showed the genetic distances among the 21 avocado and one *C. Micranthum* accessions based on the 701,352 SNPs from SLAF-seq that was inferred by UPGMA cluster analysis. The genetic distances between the Mexican accessions (Walter Hole and Duke7) and *C. micranthum* (0.56 and 0.56, respectively) were smaller than those between the Guatemalan accessions (Reed and Nabal) and *C. micranthum*(0.57 and 0.58, respectively), and those between the West Indian accessions (Pollock, Donnie, and Simmonds) and *C. micranthum* (0.59, 0.60, and 0.60, respectively) (Table S5).

**Figure 2:**
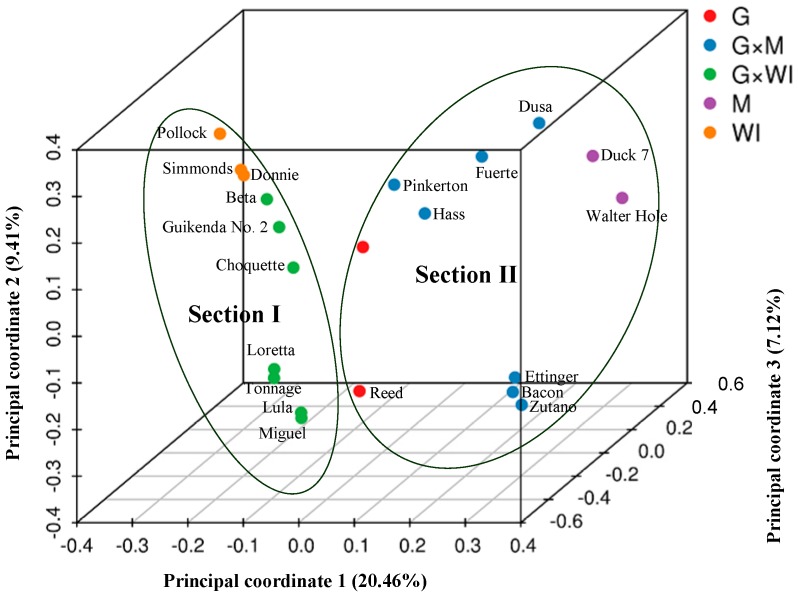
Principal coordinate analysis of the 21 avocado accessions based on 701,352 SNPs from SLAF-seq. M: Mexican, G: Guatemalan, WI: West Indian (interracial hybrids are indicated by ×).

**Figure 3:**
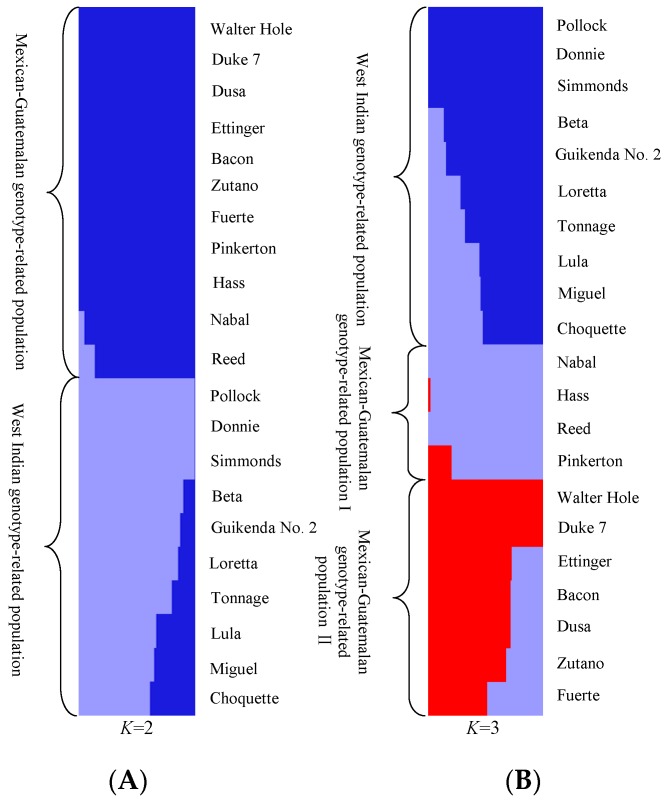
Population structure of avocado. (**A**) Population structure analysis of 21 avocado accessions under (the predicted optimal) number of populations (*K* = 2) based on 34,704 SNPs from SLAF-seq using STRUCTURE; each individual is represented by a vertical bar. (**B**) Population structure analysis of 21 avocado accessions at *K* = 3 based on 34,704 SNPs from SLAF-seq using STRUCTURE; each bar represents a single individual: blue, West Indian; purple, Guatemalan; and red, Mexican.

*PCoA* (Figure 2) and STRUCTURE output (Figure 3) produced results similar to those of the phylogenetic analysis. In *PCoA*, the three main principal coordinates explained approximately 36.99% of the total variation (20.46%, 9.41%, and 7.12% individually). The three West Indian and seven Guatemalan × West Indian hybrid accessions clustered in one group, whereas the two Mexican races, seven Guatemalan × Mexican hybrids, and two Guatemalan accessions clustered together. The two sections were obviously distinct (Figure 2). In the model-based analysis, assignment of all 21 individuals from the three ecological races and two interracial hybrids to genetic clusters using STRUCTURE revealed that the optimal number of populations (*K*) was two for genetic clusters (Figure S1). The model with *K* = 2 grouped 10 individuals into one West Indian genotype population, which was composed of three West Indian and seven Guatemalan × West Indian hybrid accessions, and the other 11 individuals together as a Mexican–Guatemalan genotype population, which was composed of the two Mexican, two Guatemalan, and seven Guatemalan × Mexican hybrid accessions (Figure 3A). With the model *K* = 3, the Mexican–Guatemalan genotype population was split into two groups. Subpopulation I included the Guatemalan and predominantly Guatemalan × Mexican hybrid accessions; subpopulation II included the Mexican and predominantly Mexican × Guatemalan hybrid accessions (Figure 3B). The genotypes of the 21 avocado accessions based on SNPs from SLAF-seq using the STRUCTURE model-based analysis (*K* = 3) are shown in Table S6. At *K* = 5, the West Indian genotype population was split into three clusters (Figure S2). Three West Indian and two Guatemalan × West Indian hybrid accessions clustered together, and four Guatemalan × West Indian hybrid accessions joined into a single cluster, and one Guatemalan accession and one Guatemalan × West Indian hybrid accession clustered together.

### 3.3. Phylogenetic Analysis Using Transcriptome SNP Data

**Figure 4:**
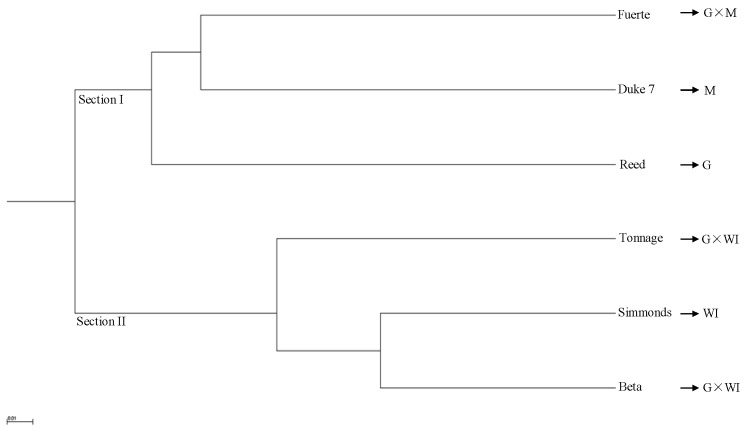
Unrooted unweighted pair-group method with arithmetic means (UPGMA) phylogenetic tree of six avocado accessions based on 65,535 SNPs from transcriptome data. M: Mexican, G: Guatemalan, WI: West Indian (interracial hybrids are indicated by ×).

The phylogeny based on 65,535 SNPs from the transcriptome data using UPGMA cluster analysis demonstrated two distinct sections that included accessions of different ecological races and interracial hybrids (Figure 4). Section I was composed of a Guatemalan × Mexican hybrid (Fuerte), and Mexican (Duke 7) and Guatemalan (Reed) accessions. Section II included a West Indian accession (Simmonds) and Guatemalan × West Indian hybrids (Beta and Tonnage). One Guatemalan × Mexican hybrid (Fuerte) clustered with a Mexican accession (Duke7), and that cluster was sister to a Guatemalan accession (Reed) (Figure 4). The genetic distances among six avocado accessions based on the 6,5535 SNPs from the transcriptome data, as determined by UPGMA cluster analysis, are provided in Table S7. The genetic distance between West Indian (Simmonds) and Guatemalan (Reed) accessions (0.39) was less than that between West Indian (Simmonds) and Mexican (Duke7) accessions (0.48) (Table S7). These transcriptome data of six avocado accessions were deposited into GenBank under accession numbers (SRX5449731–SRX5449736).

### 3.4. Genetic Diversity and Molecular Variance Analyses

When *K* = 2 genetic clusters (Figure 3A), the genetic diversity results demonstrated that the Mexican–Guatemalan genotype population possessed slightly higher *He*, *Nei*, *I*, and PIC estimates than the West Indian genotype population (Table 2), and the Mexican–Guatemalan genotype population had slightly higher genetic diversity than the West Indian genotype population. When *K* = 3 genetic clusters (Figure 3B), *He*, *Nei*, *I*, and PIC values of Mexican–Guatemalan genotype subpopulation I were the highest, followed by Mexican–Guatemalan genotype subpopulation II, and then the West Indian genotype subpopulation (Table 2). These results indicated that Mexican–Guatemalan genotype subpopulation I, which was composed of Guatemalan accessions and predominantly Guatemalan × Mexican hybrids, possessed the highest genetic diversity, whereas the West Indian genotype subpopulation, which was composed of the West Indian accessions and Guatemalan × West Indian hybrids, had the lowest genetic diversity.

The AMOVA based on 701,352 SNPs from SLAF-seq revealed a clear population separation. When only the two populations (Mexican–Guatemalan and West Indian genotype populations) were considered, 22.10% of the variation occurred among populations, and the populations considered were significantly different (*F*_ST_ = 0.221, *p*< 0.001). When the three populations were considered (Mexican–Guatemalan genotype subpopulations I and II, and the West Indian genotype subpopulation), 23.24% of the variation occurred among populations, and the populations considered were remarkably different (*F*_ST_ = 0.232, *p*< 0.001).

### 3.5. Acquisition, Verification, and Application of Race-Specific SNPs Using SLAF-seq

After the three screening steps, eight race-specific SNP loci were acquired, including three Mexican-specific, four Guatemalan-specific, and one West Indian-specific SNP locus (Table S8). Ultimately, six KASP markers were developed and used for verification, and a list of primer information is provided in Table S9. The KASP genotyping results obtained from 13 individual accessions used for SLAF-seq were consistent with our SLAF-seq results, which indicated that the SLAF-seq and SNP calling results were reliable (Table S9).

Further, six KASP markers were applied to identify the race of eight unknown race avocado accessions. The KASP genotyping results demonstrated that all eight avocado accessions were Guatemalan × West Indian hybrids (Table S9).

## 4. Discussion

In this study, the results of the three analyses performed (UPGMA cluster, *PCoA*, and STRUCTURE) based on SLAF-seq all showed the existence of two groups or populations based on botanical race: a Mexican–Guatemalan genotype population (Mexican and Guatemalan, and Guatemalan × Mexican hybrid accessions) and a West Indian genotype population (West Indian and Guatemalan × West Indian hybrid accessions). At *K* = 2, STRUCTURE demonstrated that Mexican and Guatemalan accessions clustered with the Guatemalan × Mexican hybrid accessions, whereas the West Indian accessions clustered with the Guatemalan × West Indian hybrid accessions. Chen et al. [36] and Gross-German and Viruel [13] also found that the Guatemalan and Mexican races are more closely related to each other than to the West Indian race. This was further confirmed by the UPGMA cluster results based on SNPs from transcriptomic sequencing. Similarly, AMOVA also revealed clear separation of these two populations in our study.

At *K* = 2, STRUCTURE demonstrated that seven accessions those were identified as Guatemalan × West Indian hybrids all appeared to completely cluster with the West Indian accessions rather than the Guatemalan accessions, because the proportions of West Indian genotypes of these accessions all exceeded 50% (51.85–83.06%;Table S6).Therefore, Guatemalan × West Indian hybrids integrated more with the West Indian races. At *K* = 3, STRUCTURE model-based inference also showed that the Mexican–Guatemalan genotype population could be subdivided into two subpopulations: Mexican–Guatemalan genotype subpopulations I (Guatemalan and predominantly Guatemalan × Mexican hybrid accessions) and II (Mexican and predominantly Mexican × Guatemalan hybrid accessions),which was also confirmed by the UPGMA cluster, *PCoA*, and AMOVA results based on SLAF-seq in our study. The results of this study were consistent with those of previous reports [7,8,11,12,37]. At *K* = 5, the West Indian genotype population was divided into three independent subpopulations. One major subpopulation contained all West Indian races and two Guatemalan × West Indian hybrid accessions, and another subpopulation was dominated by four accessions that were classified as Guatemalan × West Indian hybrids; this was similar to the partition of sections I-I and I-II by UPGMA cluster analysis based on SLAF-seq. Moreover, one Guatemalan × West Indian hybrid (Choquette) clustered with one Guatemalan race (Reed) into a single subpopulation. Additionally, the proportions of Guatemalan genotype in Choquette (48.15%) was the highest among the seven Guatemalan × West Indian hybrids (Table S6); therefore, Choquette was the most likely the first that admixed with the Guatemalan races and is associated with the increased of optimal *K* value compared with other Guatemalan × West Indian hybrid accessions.

UPGMA cluster analysis based on SLAF-seq clearly revealed two avocado sections. West Indian and Guatemalan × West Indian hybrid accessions, which clustered into section I, were separated from the Mexican, Guatemalan, and Guatemalan × Mexican hybrid accessions, which clustered into section II; this was further confirmed by *PCoA* and STRUCTURE analysis with *K* = 2. In section II, Mexican and predominantly Mexican × Guatemalan hybrid accessions belonged to one subsection, and Guatemalan and predominantly Guatemalan × Mexican hybrid accessions belonged to a second subsection; this was also confirmed by STRUCTURE analysis with *K* = 3. In section I, West Indian races and three Guatemalan × West Indian hybrid accessions were separated from the remaining four Guatemalan × West Indian hybrid accessions. Similarly, *PCoA* also revealed that these three Guatemalan × West Indian hybrid accessions were closer to the West Indian than the remaining four Guatemalan × West Indian hybrid accessions in section I of *PCoA*.

UPGMA cluster, *PCoA*, and STRUCTURE with *K* = 2 based on SLAF-seq all clearly separated the Mexican and Guatemalan accessions from the West Indian accessions; this finding is consistent with those of Gross-German and Viruel [13] and Chen et al. [36], who also suggested that the Guatemalan and Mexican races are more closely related to each other than to the West Indian race. Geographically and climatically, the Guatemalan and Mexican races both evolved in tropical highlands and borderline ‘cool subtropical’ areas and possess common ecological niches in the Central America highlands; alternatively, the West Indian race is adapted to tropical conditions, and evolved at low altitudes in western coastal areas of Central America [1,11].STRUCTURE analysis with *K* = 3 based on SLAF-seq further precisely differentiated the Mexican, Guatemalan, and West Indian races. These results were also confirmed in this study by the UPGMA cluster analysis based on transcriptome data. The phylogenetic analysis of the SLAF-seq and transcriptome data of the three races in this study was consistent with the previously reported genetic relationships of the Guatemalan, Mexican, and West Indian races [7,8,11,12,37].The results of this study revealed distinct phylogenetic relationships of the three ecological races and clarified the diffuse racial boundaries among these three ecological races, which supports the findings of previous studies [6,7,8,9,10,11,12,13].

The main criteria of assigning cultivar race assignation are its pedigree and horticultural characteristics. In most avocado cultivars, the pedigree is unknown or based only on the maternal tree, and race assigned based on phenotypic assessment is only tentative [11,12], because most commercial avocado cultivars are interracial hybrids [11,12,23,37]. The STRUCTURE analysis provided new insight based on abundant genotype accession data, and the genotype and racial allocation of each cultivar could be assigned based on the genotypes of the putative parental plants. The genotypes of the 21 avocado accessions based on SNPs from SLAF-seq using STRUCTURE analysis with *K* = 3 were also elucidated (Table S6). Walter Hole and Duke 7 were considered to have pure Mexican genotypes, and Fuerte, Zutano, Dusa, Bacon and Ettinger were classified as predominantly Mexican × Guatemalan hybrids, because the proportion of the Mexican genotype reached 51.35%, 66.29%, 70.00%, 70.17%, and 71.02%, respectively; their residual genome components were identified as Guatemalan. Similarly, Reed and Nabal had pure Guatemalan genotypes, and Pinkerton and Hass contained a substantial proportion of the Guatemalan genome (76.33% and 93%) and a small quantity of the Mexican genome. Hence, Pinkerton and Hass could be classified as predominantly Guatemalan × Mexican hybrids. The three accessions (Pollock, Donnie and Simmonds) were inferred to have pure West Indian genotypes. Choquette, Miguel, Lula, Tonnage, Loretta, Guikenda No. 2 and Beta seemed to be a combination of West Indian and Guatemalan gene pools and were considered as West Indian × Guatemalan hybrids; the proportions of West Indian genotype in these accessions were 51.85%, 53.80%, 54.83%, 65.77%, 80.67%, and 83.06%, respectively. Most of the 21 avocado accessions used for SLAF-seq in this study were high-quality and the cultivars are cultivated worldwide, especially Hass. Therefore, precise evaluation of the genetic backgrounds of these 21 avocado accessions has important implications for future breeding programs.

Our data provided strong evidence that the Guatemalan and Mexican races, and Guatemalan × Mexican hybrid accessions possessed higher genetic diversity than the West Indian race and Guatemalan × West Indian hybrid accessions, which was also previously noted [11,12,13]. Schnell et al. [12] found that Mexican and Guatemalan avocado accessions were highly heterozygous and heterogenous, whereas those of the West Indian race were more homozygous and homogenous. Therefore, the Mexican and Guatemalan races should attract more attention for both seedling selection and artificial hybridization in future avocado breeding programs.

## 5. Conclusions

In this study, we applied two different next-generation sequencing technologies (SLAF-seq and transcriptome) to enhance avocado germplasm classification and provided new insight into the phylogeny of the three ecological races and two interracial hybrids; additionally, we analyzed the molecular results in depth using different statistical methods (two similarity-based methods and one model-based method). We developed six race-specific KASP markers based on SNPs from SLAF-seq that successfully assigned the races of unknown avocado accessions. These data provided important information about avocado phylogenomics and will be useful for further research on avocado resource utilization and marker-assisted breeding.

## Figures and Tables

**Table 1:** Sources of the 29 avocado accessions evaluated in this study.

Accession	Origin	Source	Race	Type
Walter Hole	California, USA	GVTC, Guangxi, China	M [11]	C
Duke7	California, USA	CATAS-SSCRI, Guangdong, China	M [1,11]	RS
Nabal	Antigua, Guatemala	GVTC, Guangxi, China	G [11]	C
Reed	California, USA	CATAS-SSCRI, Guangdong, China	G [1]	C
Pollock	Florida, US	CATAS-SSCRI, Guangdong, China	WI [1]	C
Donnie	Florida, USA	CATAS-SSCRI, Guangdong, China	WI [1]	C
Simmonds	Florida, USA	CATAS-SSCRI, Guangdong, China	WI [1]	C
Bacon	California, USA	GVTC, Guangxi, China	G × M [1,11]	C
Hass	California, USA	GVTC, Guangxi, China	G × M [1,11,12,13]	C
Pinkerton	California, USA	GVTC, Guangxi, China	G × M [11,13,14,15,16,17,18,19,20,21,22,23,24,25]	C
Zutano	California, USA	GVTC, Guangxi, China	G × M [1,11]	C
Ettinger	KefarMalal, Israel	GVTC, Guangxi, China	G × M [1,11,12,13]	C
Fuerte	Puebla, Mexico	CATAS-SSCRI, Guangdong, China	G × M [1,11,12,13]	C
Dusa	Westfalia Estate, South Africa	CATAS-SSCRI, Guangdong, China	G × M [11]	RS
Miguel	Florida, USA	CATAS-SSCRI, Guangdong, China	G × WI [1]	C
Loretta	Florida, USA	CATAS-SSCRI, Guangdong, China	G × WI [1]	C
Beta	Florida, USA	CATAS-SSCRI, Guangdong, China	G × WI [1]	C
Choquette	Florida, USA	CATAS-SSCRI, Guangdong, China	G × WI [1,12]	C
Lula	Florida, USA	CATAS-SSCRI, Guangdong, China	G × WI [1,12]	C
Tonnage	Florida, USA	CATAS-SSCRI, Guangdong, China	G × WI [1]	C
Guikenda No. 2	Guangxi, China	GVTC, Guangxi, China	G × WI [26]	C
Guikenda No. 3	Guangxi, China	GVTC, Guangxi, China	Unknown	LS
Guikenda No. 4	Guangxi, China	GVTC, Guangxi, China	Unknown	LS
Guiyan No. 8	Guangxi, China	GVTC, Guangxi, China	Unknown	LS
Guiyan No. 10	Guangxi, China	GVTC, Guangxi, China	Unknown	LS
Qiongken No. 1	Guangxi, China	GVTC, Guangxi, China	Unknown	LS
Qiongken No. 2	Guangxi, China	GVTC, Guangxi, China	Unknown	LS
Daling No. 2	Guangxi, China	GVTC, Guangxi, China	Unknown	LS
Daling No. 4	Guangxi, China	GVTC, Guangxi, China	Unknown	LS

Origin: Breeding place of avocado accession, Source: Collection place of avocado accession, M: Mexican, G: Guatemalan, WI: West Indian (interracial hybrids are indicated by × LS local selection, C: commercial cultivar, RS: rootstock (commercialized clones or seedlings), CATAS-SSCRI: South Subtropical Crops Research Institute, Chinese Academy of Tropical Agricultural Sciences, GVTC: Guangxi Vocational and Technical College.

**Table 2:** Genetic diversity parameters of the different populations identified in 21 avocado accessions based on 70,1352 SNPs from SLAF-seq.

Population	Subpopulation	*Ho*	*He*	*Nei*	*I*	PIC	MAF
Population (*K* = 2)	Mexican–Guatemalan genotype-related population	0.21	0.31	0.33	0.48	0.26	0.23
	West Indian genotype-related population	0.21	0.30	0.32	0.47	0.25	0.22
Population (*K* = 3)	Mexican–Guatemalan genotype-related population I	0.29	0.39	0.48	0.58	0.31	0.31
	Mexican–Guatemalan genotype-related population II	0.25	0.33	0.37	0.50	0.27	0.24
	West Indian genotype-related population	0.21	0.30	0.32	0.47	0.25	0.22

Populations were separated based on population structure analysis (*K* = 2 and *K* = 3) in this study, *Ho*: observed heterozygosity, *He*: expected heterozygosity, *Nei*: Nei diversity index, *I*: Shannon’s information index, PIC: polymorphic information content, MAF: minor allele frequency.

## Supplementary Materials

The following are available online at https://www.mdpi.com/2073-4425/10/3/215/s1, Table S1. Summary of 22 accessions by SLAF sequencing data, Table S2. Description of the 701,352 SNPs from SLAF-seq and the genotypes of the 21 avocado and one *Cinnamomum micranthum* accession, Table S3. Summary of six accessions based on transcriptomic data, Table S4. Description of the 65,535 SNPs analyzed from transcriptome and the genotypes of the six avocado accessions, Table S5. Genetic distances among 21 avocado accessions and one *Cinnamomum micranthum* accessions based on 701,352 SNPs from SLAF-seq resulting from a UPGMA cluster analysis, Table S6. Genotypes of 21 avocado accessions based on SNPs from SLAF-seq using the STRUCTURE model-based analysis (*K* = 3), Table S7. Genetic distances among six avocado accessions based on 65,535 SNPs from transcriptome data that resulted from a UPGMA cluster analysis, Table S8. The summary of race-specific SNPs in avocado filtered from 701,352 SNPs obtained by SLAF-seq, Table S9. KASP genotyping results. Figure S1. Delta *K* values for different numbers of populations assumed in the STRUCTURE analysis. Figure S2. Population structure analysis of 21 avocado accessions at *K* = 5 based on 34,704 SNPs from SLAF-seq using STRUCTURE software.
