# Supplementary material for: Genome-Wide Assessment of Avocado Germplasm Determined from Specific Length Amplified Fragment Sequencing and Transcriptomes: Population Structure, Genetic Diversity, Identification, and Application of Race-Specific Markers
Source: Genes (Basel). 2019 Mar 13;10(3):215. doi: 10.3390/genes10030215 (PMC6471495; doi:10.3390/genes10030215)
Supplement: Supplementary file 1 [file genes-10-00215-s001.zip › Figure S1. Delta K values for different numbers of populations assumed in the STRUCTURE analysis.doc]

**Figure S1.** Delta *K* values for different numbers of populations assumed in the STRUCTURE analysis.
