# Supplementary material for: Genome-Wide Assessment of Avocado Germplasm Determined from Specific Length Amplified Fragment Sequencing and Transcriptomes: Population Structure, Genetic Diversity, Identification, and Application of Race-Specific Markers
Source: Genes (Basel). 2019 Mar 13;10(3):215. doi: 10.3390/genes10030215 (PMC6471495; doi:10.3390/genes10030215)
Supplement: Supplementary file 1 [file genes-10-00215-s001.zip › Figure S2. Population structure analysis of 21 avocado accessions at K = 5 based on 34,704 SNPs from SLAF-seq using STRUCTUER software.doc]

**Figure S2.** Population structure analysis of 21 avocado accessions at *K* = 5 based on 34,704 SNPs from SLAF-seq using STRUCTURE software.
